# Framing effects reveal discrete lexical-semantic and sublexical procedures in reading: an fMRI study

**DOI:** 10.3389/fpsyg.2015.01328

**Published:** 2015-09-23

**Authors:** Laura Danelli, Marco Marelli, Manuela Berlingeri, Marco Tettamanti, Maurizio Sberna, Eraldo Paulesu, Claudio Luzzatti

**Affiliations:** ^1^Psychology Department, University of Milan-BicoccaMilan, Italy; ^2^NeuroMI -Milan Center for NeuroscienceMilan, Italy; ^3^Center for Mind/Brain Sciences, University of TrentoRovereto, Italy; ^4^Division of Neuroscience and Department of Nuclear Medicine, San Raffaele Scientific InstituteMilan, Italy; ^5^Neuroradiology Department, Niguarda Ca' Granda HospitalMilan, Italy; ^6^fMRI Unit, IRCCS GaleazziMilan, Italy

**Keywords:** reading, fMRI, list-manipulation paradigm, dual-route model, lexical-semantic procedure, sublexical procedure, multi-voxel pattern analysis (MVPA)

## Abstract

According to the dual-route model, a printed string of letters can be processed by either a grapheme-to-phoneme conversion (GPC) route or a lexical-semantic route. Although meta-analyses of the imaging literature support the existence of distinct but interacting reading procedures, individual neuroimaging studies that explored neural correlates of reading yielded inconclusive results. We used a list-manipulation paradigm to provide a fresh empirical look at this issue and to isolate specific areas that underlie the two reading procedures. In a lexical condition, we embedded disyllabic Italian words (target stimuli) in lists of either loanwords or trisyllabic Italian words with unpredictable stress position. In a GPC condition, similar target stimuli were included within lists of pseudowords. The procedure was designed to induce participants to emphasize either the lexical-semantic or the GPC reading procedure, while controlling for possible linguistic confounds and keeping the reading task requirements stable across the two conditions. Thirty-three adults participated in the behavioral study, and 20 further adult participants were included in the fMRI study. At the behavioral level, we found sizeable effects of the framing manipulations that included slower voice onset times for stimuli in the pseudoword frames. At the functional anatomical level, the occipital and temporal regions, and the intraparietal sulcus were specifically activated when subjects were reading target words in a lexical frame. The inferior parietal and anterior fusiform cortex were specifically activated in the GPC condition. These patterns of activation represented a valid classifying model of fMRI images associated with target reading in both frames in the multi-voxel pattern analyses. Further activations were shared by the two procedures in the occipital and inferior parietal areas, in the premotor cortex, in the frontal regions and the left supplementary motor area. These regions are most likely involved in either early input or late output processes.

## Introduction

In the study of reading, dual-route models (Coltheart et al., 1980, 2001) have been very influential in both experimental psychology and neuropsychology. These models assume that a string of letters can be processed by two procedures. One procedure is based on a GPC route to generate individual sounds and the assembled phonology represented by the whole string. The other procedure is based on a lexical, or a lexical and semantic, route that is initially activated in the form of an abstract word representation in the *orthographic input lexicon*. This would then activate the corresponding conceptual representation and the phonological word form. These two processing routes have been assumed to run in parallel, and in principle, any orthographic input representation triggers the activation of both streams. However, this does not necessarily mean that all stimuli can be read correctly along both routes. Indeed, the involvement of either route in processing specific stimuli leads to erroneous (or even impossible) outcomes. Pseudowords (e.g., *sploice*) cannot be read via the lexical route because they lack a lexical representation, and irregular words (e.g., *yacht*) are doomed to incorrect readings (regularizations) when they are processed with a GPC procedure (*yacht* read as /jt/). The two processing-routes hypothesis (the dual-route model) has been studied extensively in cognitive neuropsychology. Patients who are impaired in pseudoword reading and whose performance on words was flawless (phonological dyslexia; Beauvois and Derousné, 1979; Shallice and Warrington, 1980; Coltheart, 1996), and patients who correctly read pseudowords and regular words but fail when trying to read irregular words (surface dyslexia; Marshall and Newcombe, 1973) represent a double dissociation in support of the dual-route hypothesis. The former type of dyslexia can be explained as a consequence of specific damage to the GPC procedure, and the latter is interpreted in terms of an impairment of the lexical route^1^. The success of the dual-route approach in explaining neuropsychological impairment has made it a reference model in the field and an important theoretical framework for clinical assessment.

Anatomical investigation in patients with specific forms of dyslexia suggests that the cognitive procedures that are involved in either processing route could be associated with two anatomically segregated processing streams for reading abilities. Poor reading of pseudowords is usually associated with temporo-parietal and left frontal lesions (e.g., Friedman and Kohn, 1990; Friedman, 1996; Patterson et al., 1996; Fiez et al., 2006; Sato et al., 2008; Rapcsak et al., 2009), and an impairment in reading irregular words is often observed with left anterolateral temporal lobe damage (e.g., Patterson and Behrmann, 1997; Wilson et al., 2009; see also Ripamonti et al., 2014 for a voxel-based symptom mapping analysis of 59 dyslexic patients). However, the concept of independent and segregated networks that are associated with each reading route is not unequivocally accepted in the literature for both empirical and theoretical reasons. From an empirical point of view, neuropsychological results are not conclusive because most phonological and surface dyslexic patients suffer from extensive and heterogeneous lesions that make it difficult to establish well-localized functional anatomical correlations. Behavioral deficits can also occur due to either a lesion of a specific brain region or an anatomical disconnection between cerebral regions.

The dual-route model is only one of the theoretical frameworks that have been proposed in the literature to explain reading processes. The most successful alternative to the dual-route model, the connectionist model (or *triangle model*; Seidenberg and McClelland, 1989; Plaut et al., 1996; Seidenberg, 2005) suggests a strongest emphasis on the orthography-to-semantics-to-phonology pathway for irregularly spelled words and on orthography-to-phonology processes for pseudowords (for a functional anatomical demonstration see Mechelli et al., 2005). Crucially, this model does not postulate a separate, lexical non-semantic route for reading.

### Functional imaging contributions to the identification of specific pathways for reading: The impregnable fortress of the dual-route pathway

There are several reasons why the imaging literature has failed to provide convincing evidence of dissociable neural systems for the sublexical and lexical routes (see Cattinelli et al., 2013; Taylor et al., 2013). Many strategies have been adopted. One experimental strategy has been to manipulate task demands rather than stimuli (Rumsey et al., 1997; Cappa et al., 1998; Mummery et al., 1998; Booth et al., 2002). The assumption made in these studies is that very similar items may be processed differently by varying the specific task demands. A classical implementation of this rationale has been the adoption of semantic as opposed to phonological judgment tasks for the same stimuli. The results of these two approaches would provide information about the areas that are involved in the lexical route or in the GPC route, respectively (Price et al., 1997; Rumsey et al., 1997; Mummery et al., 1998; Booth et al., 2002). This approach is complicated by the difficulty of controlling for the activation of semantic representations. A further problem in the task-demand manipulation approach is that certain cognitive tasks, such as phonological or semantic awareness tasks, tap into high-level cognitive layers that are associated with the decision-making processes and the selection of relevant information that suggests which cognitive judgments are to be made. It is plausible that this type of manipulation will strongly affect neural activation as well (see Table 6 in Cattinelli et al., 2013).

Another popular approach has been to use route-specific sets of stimuli. English orthography is an ideal test-bed because of its many orthographic irregularities. It has been assumed that *route-specific* sets of items would activate only those areas that are associated with a specific procedure. For example, pseudowords would emphasize the *GPC areas*, and irregular words would emphasize areas that are involved in the lexical procedure (see illustrative examples and reviews in Fiez and Petersen, 1998; Hagoort et al., 1999; Paulesu et al., 2000; Mechelli et al., 2003; Ino et al., 2009; Levy et al., 2009; Price, 2012; Cattinelli et al., 2013). Data derived from this approach are in some cases contradictory. For example, several fMRI studies reported stronger activation of the left occipito-temporal junction (Paulesu et al., 2000; Xu et al., 2001) and of the left inferior temporal cortex (Fiez et al., 1999; Paulesu et al., 2000) during pseudoword reading compared with word reading, which would suggest involvement of these areas in sublexical processing or the contribution of larger-grained representations to pseudoword reading (see Cattinelli et al., 2013). On the contrary, some studies reported a stronger activation of these same areas when reading words rather than pseudowords (Cappa et al., 1998; Hagoort et al., 1999), which would suggest involvement of these regions in written word processing.

Furthermore, this approach is prone to possible confounds (e.g., familiarity with the orthographic string, the role of variables such as word frequency, or imageability), and it strongly depends on the assumption that brain regions that are specifically involved in a given procedure (e.g., a GPC-specific region) would be functionally silent when reading stimuli that are preferentially processed by the alternative procedure. There is evidence, however, that this might not be the case: for example, the assumption that irregular > regular words would activate the lexical route and regular > irregular words would activate the GPC route is not valid, as both types of stimuli actually activate both routes (although only one is functionally relevant, see Coltheart et al., 2001; Taylor et al., 2013).

Recently, the PET/fMRI literature has been reviewed in two meta-analyses (Cattinelli et al., 2013; Taylor et al., 2013) to address this issue. Both Cattinelli et al. (2013) and Taylor et al. (2013) found evidence for greater activity during pseudoword than word reading (which should reveal activity in brain regions that are involved in spelling-to-sound conversion) in the bilateral parietal cortex and the left posterior occipito-temporal cortex. Moreover, both studies found evidence for greater activity for word than pseudoword reading (which should reveal activity in brain regions that are involved in lexical/semantic processing) in the left angular gyrus, left anterior fusiform gyrus, and left middle temporal gyrus.

### Rationale and aim of the present study

The aim of this study was to challenge the *dual-route anatomical fortress*. We capitalized on previous evidence that suggests that it is possible to influence sequential single-word reading strategies by manipulating the item lists either by employing separate lists for different item types or by mixing different types of stimuli, e.g., pseudowords and words, within the same list (Baluch and Besner, 1991; Monsell et al., 1992; Tabossi and Laghi, 1992; Lupker et al., 1997; Zevin and Balota, 2000; Decker et al., 2003; Reynolds and Besner, 2005; Kinoshita and Lupker, 2007; Kang et al., 2009; Paizi et al., 2010; see Traficante and Burani, 2014 for a review). The data seem to support the assumption that manipulation of an experimental list may induce preferential recruitment of either lexical or sublexical strategies: a reading task where regular words are mixed with irregular words might lead to intensification of the lexical process, while a condition where regular words are mixed with pseudowords might emphasize the sublexical route (the route emphasis hypothesis; Monsell et al., 1992; Reynolds and Besner, 2005). However, some authors proposed an alternative interpretation of these effects, which suggests that the onset of the verbal response in pure and mixed lists could be modulated by the specific demand that is imposed by the item to be pronounced (e.g., its phonological and articulatory aspects, and whether it is more or less frequent). According to this hypothesis, reading pace would be determined by item “difficulty” and would reflect the participant's attempt to strike a balance between reading accuracy and reading speed (the time-criterion hypothesis; Lupker et al., 1997; Kinoshita and Lupker, 2007; Kang et al., 2009).

Interestingly, although they originate from the same behavioral evidence, these two interpretations result in opposite neurofunctional predictions. The route emphasis hypothesis predicts that the adoption of list manipulation would result in the recruitment of different neural patterns in response to different reading procedures, while the time-criterion hypothesis predicts that the adoption of different reading items, even if they elicit a same reading procedure, would be associated with different neural activations. In particular, according to the time-criterion hypothesis, we should be able to detect between-items differences in those brain regions that are typically associated with task demand (Bedny et al., 2007; Berlingeri et al., 2008). Item demand may depend on early visual-orthographic features, on phonological-articulatory complexity, on psycholinguistic aspects or on a combination of these different levels.

In light of these considerations, our approach was to embed disyllabic real words (target words) in a frame of non-target stimuli, which would lead participants to place more emphasis on either the lexical or the sublexical reading procedure. Irregular words^2^ would emphasize the lexical procedure, and pseudowords would emphasize the GPC reading procedure. We used two classes of irregular lists (trisyllabic words with unpredictable stress position, loan words that are largely employed in the Italian language, e.g., “computer”) to allow us to generalize our findings beyond one single class of stimuli. Moreover the use of two experimental conditions, one for loanwords and the other for trisyllabic words, allowed us not only to elicit the lexical-semantic reading strategy (or the GPC reading procedure with the pseudoword frames), but also to address the time-criterion hypothesis. Indeed, even if our fMRI manipulation paradigm was designed to specifically address the route-emphasis hypothesis and to disentangle the neurofunctional correlates of the lexical and sublexical routes while controlling all possible lexical and experimental confounds, the concept of item demands allowed us to assess the time-criterion hypothesis by looking for a possible interaction between experimental conditions and lexicality in the fMRI data.

In other words, if a gradient of difficulty between different filler lists would actually occur (i.e., pseudowords with CV structure easier to read than pseudowords with complex consonant clusters, and words with CV structure easier to read than loanwords) and this difficulty effect would influence the reading speed of target words, as assumed by the time-criterion hypothesis, an interaction effect between session and lexicality should emerge at anatomo-functional level. Whereas, if no interaction effects would emerge from fMRI data, we may infer that the list manipulation actually induces different reading procedures, thus supporting the route-emphasis hypothesis.

Finally, it is worthy to note that the target stimuli for the analyses of the hemodynamic responses were always disyllabic words. These were accurately matched for a number of psycholinguistic properties, such as word frequency, phonological complexity, orthographic neighborhood size, imageability, and beginning phonemes. With this new experimental paradigm, we tried to find evidence for process-specific areas and for brain regions that are shared by the two reading procedures, and simultaneously we tried to control for possible psycholinguistic and task-related confounds.

## Materials and methods

### Behavioral study

#### Participants

Thirty-three healthy young adult participants (17 M/16 F; mean age = 28.6 ± 4.4 years) took part in the study. The participants had no history of neurological and psychiatric disorders. They all had normal cognitive development, normal or corrected-to-normal vision, and normal language and reading skills.

#### Stimuli

Regular disyllabic Italian words (target stimuli) were embedded in a frame of either irregular words or pseudowords (fillers). There were four lists, and each comprised 20 disyllabic Italian words. All experimental words were nouns with a consonant-vowel (CV) structure. The four lists were matched for word frequency, orthographic neighborhood size, imageability, and beginning phonemes. Word frequency and orthographic neighborhood size measures were obtained from the COLFIS corpus (Laudanna et al., 1995). Imageability of word stimuli was evaluated by ten graduate students in a preliminary study. These student participants were asked to rate each word on a seven-point rating scale that ranged from “very difficult to imagine” to “very easy to imagine,” which described the extent to which the concept underlying the word was associated with a mental image. Distribution-based matching was performed because psycholinguistic effects are not always linear (e.g., Bien et al., 2005). The distribution of the variables in the four experimental lists did not differ (Kolmogorov-Smirnov tests: n.s.).

Each experimental list was randomly associated with a list of fillers. The purpose of the filler lists was to prime a prevalent use of either the lexical-semantic reading procedure or the GPC reading procedure. The lexical procedure was elicited through the use of both trisyllabic Italian words and foreign loanwords. The first list of lexical fillers contained 30 trisyllabic words, in which lexical stress was either on the penultimate syllable (15 words, e.g., *parola*, /pa^ɪ^rola/, word) or on the antepenultimate syllable (15 words, e.g., *tavolo*, /^ɪ^tavolo/, table). The second list of lexical fillers comprised 20 English loanwords (e.g., *barbecue*, /^ɪ^bɑ:bɪkju:/) and 10 French loanwords (e.g., *beige*, /bε:ʒ/) that are currently used in Italian but are not readable when following regular GPC rules. GPC reading was elicited by means of pseudowords. Two lists of pseudowords were created to be as orthographically similar as possible to the corresponding lexical filler lists. The first list contained 30 trisyllabic pseudowords with a CV structure (e.g., *dogore*), which matched the filler list of trisyllabic words, and the second list included 30 pseudowords that contained consonant clusters (e.g., *cimpelte*), which matched the filler list of loanwords. The length of pseudowords was matched with the length of familiar words in the corresponding filler lists.

See Appendix 1 for a complete list of the target and filler items.

#### Experimental procedure

Target disyllabic words were presented with filler stimuli and presumably elicited a reading process that followed either the lexical-semantic procedure (lexical frame) or the GPC procedure (pseudoword frame). Targets were presented in ten-item blocks that had either a lexical-semantic or a pseudoword frame. The target-word rate was 4/10 for each block. Targets and fillers were presented in semi-randomized order, i.e., in “mini-blocks” that reflected an alternating sequence of 2–3 fillers and 1–2 targets.

Lexical-semantic and pseudoword frame conditions were alternated and counterbalanced across participants. Each frame condition was preceded by a baseline sequence that comprised strings of lines that were oriented differently and were matched with the orthographic stimuli for length, numbers of lines, and visual angle.

**Figure 1:**
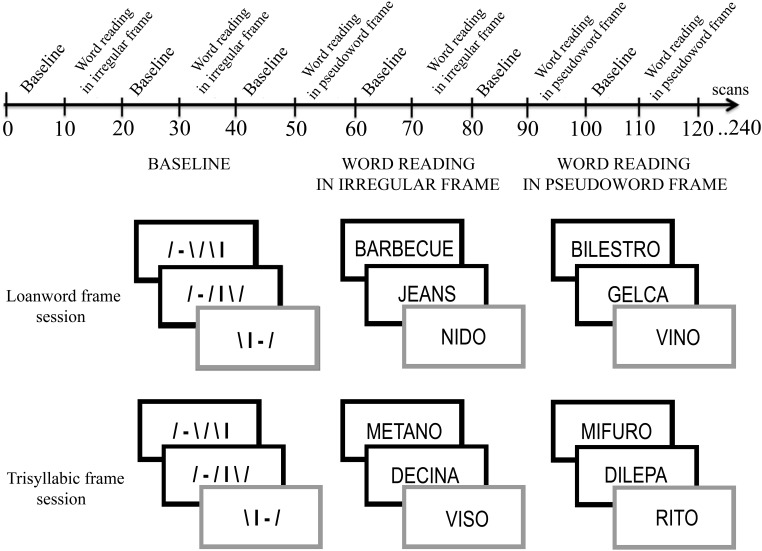
**Schematic representation of the time line of tasks**.

There were two separate sessions. In the first session (*loanword-frame session*), the disyllabic target words were embedded in filler lists that were made up of either loanwords or pseudowords that contained consonant clusters (CC). In the second session (*trisyllabic-frame session*), the disyllabic target words were embedded in filler lists that were formed of either trisyllabic Italian words or pseudowords with a CV structure. Participants were randomly assigned to one of the two tasks (Figure 1).

All participants in the behavioral study read an additional list of 40 CV-disyllabic target words (block condition).

Therefore, each participant performed only one reading session (a “loanword session” or a “trisyllabic session”) because the remaining stimuli were used in the “block condition.”

All the stimuli (font: Arial; size: 42; color: black) were displayed in the center of a computer screen on a white background by means of E-Prime software (Psychology Software Tools Inc., Pittsburgh, PA). Participants were instructed to read the letter strings aloud and to press a key on a serial response box for each string of lines. Reading accuracy and voice-onset time (VOT) were recorded. Stimuli remained on the screen until the participant responded. The inter-stimulus interval (ISI) was 1500 ms.

### fMRI study

#### Participants

A new sample of 20 normal young right-handed adult participants (10 M/12 F; mean age = 24.1 ± 4.4 years) with advanced education (mean education = 15.7 ± 1.7 years) took part in the fMRI study.

All were native Italian speakers with no history of neurological and psychiatric disorder. They all had normal cognitive development, normal or corrected-to-normal vision, and normal language and reading skills.

Informed consent was obtained from all participants prior to the scanning session.

#### Experimental procedure

The fMRI study replicated the behavioral experiment as closely as possible.

The fMRI design was based on alternating 30-s baseline blocks (ten blocks for each session) and experimental blocks. In both sessions, the baseline stimuli were strings of lines with different orientation that were matched with the experimental stimuli for length, number of components, and visual angle. The experimental stimuli were the same words, loanwords and pseudowords that were used in the behavioral study (see Figure 1).

In the first session *(loanword-frame session)*, the target disyllabic words were alternated with both loanwords (a *loanword frame)* and trisyllabic pseudowords that contained CC (a CC-*pseudoword frame*). In the second fMRI session *(trisyllabic-frame session)*, the disyllabic words were alternated with both trisyllabic Italian words (a *CV*-*trisyllabic-word frame*) and trisyllabic pseudowords with a CV structure (a *CV-pseudoword frame*). The target-word rate was 4/10 for each block in both sessions.

During the fMRI sessions, stimuli were projected from a PC that was located outside the MR room and was connected by optical fibers to dedicated goggles (Visuastim XGA, Resonance Technology, www.mrivideo.com) using Presentation 11 software (Neurobehavioral Systems, Inc., Albany, CA). Specific corrective lenses were used in the scanner for volunteers with known refraction deficits.

Participants were exposed to each stimulus for 1500 ms in each session. Stimuli were shown in the center of a white screen. The interstimulus interval (ISI) was randomly selected in a time-window of 1200–1800 ms to avoid habituation to the BOLD signal. Participants were exposed to a white screen during the ISI.

Participants were instructed to read words, loanwords, and pseudowords silently to avoid artifacts that would be caused by mouth and head movements. They were asked just to look at the pattern of lines for the baseline task. Participants were also instructed to press a button after each stimulus. Half of the participants pressed the key-button with the right index finger, the other half with the left index finger.

Sessions were presented in a counterbalanced order across participants.

Unlike the behavioral study, all of the participants in the fMRI study performed both the loanword and the trisyllabic sessions, and none performed the block condition.

#### Image acquisition

For each participant, 214 fMRI cerebral scans for each reading task were collected using an echo-planar gradient-echo pulse sequence (EPI; Ogawa et al., 1992), T2^*^ weighted, with a 1.5 T GE-Signa scanner (Slice thickness = 4 mm; Flip angle 90°; TE = 60 ms, TR = 3000 ms, FOV = 240 × 240 mm; matrix = 64 × 64).

#### fMRI analyses

The fMRI analyses were performed using the SPM8 software (Welcome Department of Imaging Neuroscience, University College, London). The fMRI images that were collected for all participants were realigned to remove movement artifacts and then were normalized in the MNI-space. Images were then convolved in space with a three-dimensional isotropic Gaussian kernel (10 mm FWHM) to improve the signal-to-noise ratio. A subject-by-task first level analysis was performed after this standard pre-processing step. There were thus two fixed-effect analyses for each participant. The BOLD signal was convolved using the standard hemodynamic response function (HRF) and modeled according to an event-related design (Worsley and Friston, 1995). The event-related matrix was designed to isolate the hemodynamic response that was elicited by reading fillers and by reading targets within each list. Finally, for each subject we estimated the four condition-specific effects of interest: (i) *target reading* > *baseline* in the *loanword frame*, (ii) *target reading* > *baseline* in the *CC-pseudoword frame*, (iii) *target reading* > *baseline* in the *CV-trisyllabic-word frame*, (iv) *target reading* > *baseline* in the *CV-pseudoword frame*. We therefore obtained four “contrast images,” i.e., four maps that included the effect of interest per voxel of the brain for each participant. These contrast images were entered into a random-effect analysis that conformed to a general linear model (GLM, Holmes and Friston, 1998; Penny and Holmes, 2004).

A 2^*^2 second-level ANOVA that had four within-subject conditions (that corresponded to the effects that were described above) was designed and estimated. The factors were *frame session* (loan vs. tri-syllabic) and *lexicality* (word-frame vs. pseudoword-frame).

Because we were interested in disentangling the areas of the reading neural network that are specific to the lexical-semantic procedure from those areas that are specific to the GPC procedure, the ANOVA was explicitly masked by the neural network that was associated with word and pseudoword reading (*p* < 0.001), as described in one of our previous papers (Danelli et al., 2013), i.e., the ANOVA was computed only in the voxels that belonged to the mask.

This allowed us to focus on brain regions that are typically responsive to reading, i.e., the responses should be positive relative to a minimal baseline for word and pseudoword reading, excluding any negative BOLD response within this constrained mask. Moreover, this allowed us to reduce the problem of multiple comparisons (41.0 resels). The mask included the prefrontal and frontal cortex, bilaterally, the insulae, a large part of both temporal lobes, the left parietal lobule, and the primary and secondary visual cortex, bilaterally (see Appendix 2).

We first looked for brain regions that showed a significant interaction effect between experimental condition and frame (*p* < 0.001).

This experimental paradigm helped us to test the neurofunctional correlates of the two reading procedures, while controlling for all psycholinguistic confounds. Indeed, we were comparing the BOLD signal associated with reading “MULO” (mule) with the BOLD signal that is associated with reading “RANA” (frog), i.e., items that are psycholinguistically almost identical.

For this reason, we used both a direct-comparison approach with low threshold (*p* < 0.05) and a less conservative approach based on spatial inference rather than on specific voxel-wise inference. To fully achieve this aim we cleaned the spurious pattern by excluding the activation of “non-interest” by means of an exclusive masking procedure (which has been successfully employed in a number of previous fMRI studies: Pochon et al., 2002; Uncapher et al., 2006; Fliessbach et al., 2007; Danelli et al., 2013).

From the univariate second-level analysis, we extracted:

1. The direct comparisons between target items in lexical-semantic and sublexical frames (lexical > GPC and GPC > lexical) were computed (*p* < 0.05; spatial threshold = 10 voxels).
2. Lexical-semantic effect: this was computed as a main effect of the target-word reading in the loanword frame and in the CV-trisyllabic-word frame (*p* < 0.001 uncorrected). This analysis was exclusively masked so that voxels “belonging” to the GPC procedure were excluded from the test. The map for the exclusive mask was generated by using a low threshold (*p* < 0.05 uncorrected). This ensured that the analysis did not consider voxels showing weakest trends for activations in the GPC condition.
3. GPC effect: this was computed as a main effect of the target reading in the CC-pseudoword frame and in the CV-pseudoword frame (*p* < 0.001 uncorrected). An exclusive masking procedure was used as above but this exclusive mask was derived from the lexical-semantic condition.
4. Conjunction of the lexical-semantic and GPC effects: the GPC effect and the lexical-semantic effect were entered in a conjunction analysis (Friston et al., 1999; Worsley and Friston, 2000) to identify the brain regions that are commonly activated by both the lexical and the GPC reading procedures (*p* < 0.001). This conjunction was computed as in the univariate analysis adopting a conservative conjunction approach based on minimum statistics procedure (Nichols et al., 2005).

Finally, in order to test whether the isolated networks corresponding to the latest three effects would represent good classifier models of the fMRI images associated with the target reading performance in the lexical or in the sublexical frame, we implemented three multivariate classification analyses, by means of multi-voxel pattern analysis (MVPA), using the PyMVPA 2.2 toolbox (www.pymvpa.org; Hanke et al., 2009). These analyses were implemented in order to support the hypothesis that the exclusive masking could be a valid approach, even if less conservative than a direct comparison method. To this end, we repeated the SPM8 univariate first-level analysis on realigned and spatially normalized, but spatially unsmoothed fMRI data. We computed spmT maps associated with the four condition-specific effects of interest (see the univariate analysis), which were then used for the MVPA (Misaki et al., 2010).

MVPA was performed on the data of 20 subjects. We trained the linear support vector machine classifier algorithm implemented in PyMVPA with a leave-one-subject-out cross validation procedure, using for each iteration the spmT maps of 19 subjects, and then testing the classification accuracy on the spmT maps (2 for the lexical and 2 for the sublexical condition-specific effects) of the 20th subject. In particular, we ran three different independent multivariate classification analyses using as inclusive mask, respectively, the lexical-semantic, the GPC, and the conjunction effects described above, although at a less conservative significance threshold. These three analyses were run to specifically test the following scenarios:

1. if the lexical-semantic mask, extracted by means of the massively univariate analysis, actually represented the pool of brain regions exclusively associated with the lexical-semantic reading procedure, then the classifier should be able to accurately distinguish between the lexical-semantic and the GPC spmT maps;
2. similarly, if the GPC mask extracted from the standard random effect analysis represented the pool of brain regions associated with reading by the GPC procedure, then once again the MVPA should accurately distinguish between the two types of spmT maps;
3. on the contrary, if the mask extracted from the conjunction effect analysis actually represented the pool of brain regions that are commonly activated by the two procedures, then the MVPA procedure should fail to distinguish between the lexical semantic and the GPC spmT maps.

As for the latter scenario, we further considered whether the MVPA could be a more sensitive approach than the univariate approach, and detect any spatially restricted patterns within the conjunction effect mask, that could distinguish between the lexical semantic and the GPC spmT maps, in spite of a failure at the whole-mask level. To this purpose, we employed recursive feature elimination (Hanson and Halchenko, 2008). Recursive feature elimination was performed strictly within the training partitions, by iteratively eliminating the less sensitive 50% of voxels, and then selecting the reduced brain voxel partition having the greatest sensitivity.

## Results

### Behavioral data

The accuracy of all participants was at ceiling for the reading tasks that were performed outside the scanner. VOTs (log-transformed) were analyzed for target words only. Data were trimmed on the basis of the visual inspection of QQ-plots. Datapoints that clearly deviated from a Gaussian distribution (i.e., VOTs that were shorter than 200 ms and longer than 950 ms) were removed.

To account for the non-independence of observations in the dataset, results were analyzed using a mixed-effects model (Baayen et al., 2008) that included random intercepts for items and participants. Outlier datapoints were identified and removed using 2.5 SD of the model residuals as a criterion. Degrees of freedoms were estimated following the method proposed by Satterthwaite (1946).

Data analysis showed a significant main effect of the list (GPC vs. lexical) [*F*_(1, 75.31)_ = 4.97; *p* = 0.0287]. Participants were significantly faster when reading disyllabic target words that were embedded in a lexical filler list (mean = 469 ms, SEM = 2.41) than when reading disyllabic target words embedded in a GPC filler list (mean = 482 ms, SEM = 2.38). Neither the interaction between list and task [*F*_(1, 75.31)_ = 0.69; *p* = 0.4081], nor the main effect of task [*F*_(1, 33.67)_ = 0.37; *p* = 0.5442] were significant.

A second mixed-effects model that also included random slopes for participants was estimated in order to provide indirect evidence that the observed pattern of results (included the absence of behavioral differences between similar frames) depend on participants recruitment biases. The predictions were confirmed: participants were significantly faster [*F*_(1, 47.97)_ = 7.41; *p* = 0.0089] when reading disyllabic target words that were embedded in a lexical filler list than when reading disyllabic target words embedded in a GPC filler list, and the interaction between list condition and task was not significant [*F*_(1, 47.97)_ = 1.14; *p* = 0.2908]. Indeed, the inclusion of the random slopes did not significantly improve model fit [$X_{\left(6\right)}^{2}=1.95$; *p* = 0.924], indicating that the associated parameters are not justified by the additional amount of explained variance.

Finally, a further analysis contrasted the responses to the different frame conditions with the responses to the same item in a block design. Participants were significantly faster when reading disyllabic words in a block condition (mean = 458 ms, SEM = 1.75) than in either the lexical [*t*_(132.61)_ = 2.42; *p* = 0.0166] or the GPC filler list [*t*_(133.13)_ = 3.24; *p* = 0.0014].

### fMRI data: Univariate analyses

No interaction effects emerged from the analyses. This result confirmed the absence of neural differences between either the two lexical frames or the two sublexical frames and justified the evaluation of lexical and sublexical frame effects using t-linear contrasts.

#### Lexical-semantic effect

##### Direct comparison approach (lexical>GPC)

An increased activation was observed in the lexical-semantic frame rather than in the GPC frame at the level of the left hemisphere, and in particular, in the inferior frontal gyrus, bilaterally, in the left precentral and postcentral gyri, in the left superior parietal lobule, in the left superior and middle temporal pole, in the left superior and middle temporal gyrus, in the left hippocampus, in the left inferior occipital gyrus, in the calcarine cortex, in the lingual gyrus and in the cerebellum. Right activations were observed in the superior temporal pole, in the middle temporal gyrus, in the inferior occipital gyrus, in the calcarine cortex and in the cerebellum (Table 1A).

**Table 1:** **Brain regions that are significantly activated in direct comparisons between lexical-semantic and sublexical frames (***p*** < 0.05; spatial threshold = 10 voxels)**.

**Brain regions**	***MNI coordinates***							
	**Left hemisphere**				**Right hemisphere**			
	***x***	***y***	***z***	***Z-score***	***x***	***y***	***z***	***Z-score***
**(A) LEXICAL EFFECT** > **GPC EFFECT**								
Inf. frontal gyrus, pars orbitalis	−52	38	−6	3.07				
	−52	40	−2	2.92				
Inf. frontal gyrus, pars triangularis					58	26	2	1.94
Inf. frontal gyrus, pars opercularis	−54	12	6	2.19	52	20	14	2.18
Precentral gyrus	−48	−4	42	2.71				
	−46	−4	46	2.64				
Postcentral gyrus	−54	−14	48	2.94				
	−46	−8	48	2.74				
Sup. parietal lobule	−38	−68	56	3.37	40	18	−22	2.59
Sup. temporal pole	−30	10	−26	3.04				
Mid. temporal pole	−42	16	−26	3.28				
Sup. temporal gyrus	−64	−48	14	2.43				
	−58	−42	14	1.98				
Mid. temporal gyrus	−56	−52	2	2.04	68	−36	4	3.02
	−60	−54	2	1.97	66	−32	2	2.62
Hippocampus	−24	−4	−24	2.81				
Inf. occipital gyrus	−34	−84	−12	2.35	40	−78	−12	2.01
	−36	−88	−8	2.09				
Calcarine cortex	−2	−86	8	2.70	4	−88	10	2.72
	−10	−92	−10	2.10	4	−86	14	2.47
Lingual gyrus	−20	−86	−16	2.15				
	−10	−86	−12	1.94				
Cerebellum	0	−46	−8	3.13	16	−80	−22	3.05
Cerebellum	−14	−84	−18	2.30	22	−82	−24	2.91
**(B) GPC EFFECT** > **LEXICAL EFFECT**								
Mid. frontal gyrus, pars orbitalis	−28	44	−12	2.54				
Inf. frontal gyrus, pars orbitalis	−32	36	−8	1.83				
	−36	36	−6	1.82				
Hippocampus	−26	−28	−4	1.98				
Inf. parietal lobule	−44	−40	40	2.27				
	−42	−40	44	2.19				
Fusiform gyrus	−38	−44	−20	2.00				
	−38	−48	−18	1.80				

##### Exclusive masking approach

**Figure 2:**
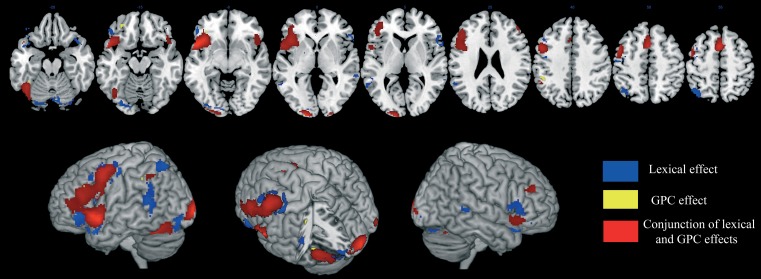
**Brain activation data**. The cerebral areas that are specifically associated with lexical processing (in blue), sublexical processing (in yellow), and with both reading procedures (in red) are displayed on an anatomical template image (the “ch2better” template image in MRICron; Rorden and Brett, 2000).

A significant activation was found in the left supplementary motor area (SMA), in the left middle frontal gyrus, in the inferior frontal gyrus, bilaterally, in the left precentral and postcentral gyri, in the left superior parietal lobule, in the left intraparietal sulcus, in the superior temporal pole, bilaterally, in the left superior temporal gyrus, in the middle temporal gyrus, bilaterally, in the left hippocampus, in the left fusiform gyrus, in the left middle occipital gyrus, in the inferior occipital gyrus, bilaterally, in the left V1, in the left lingual gyrus and in the cerebellum, bilaterally (Table 2A and areas in blue in Figure 2).

**Table 2:** **Brain regions that are significantly activated in association with either the lexical effect, the GPC effect, or the commonalities between target-word reading in the lexical and target-word reading in the sublexical frames**.

**Brain regions**	***MNI coordinates***							
	**Left hemisphere**				**Right hemisphere**			
	***x***	***y***	***z***	***Z-score***	***x***	***y***	***z***	***Z-score***
**(A) LEXICAL EFFECT**								
SMA	−10	12	48	3.14				
Mid. frontal gyrus	−44	20	42	3.53				
	−40	10	56	3.25				
Inf. frontal gyrus, pars orbitalis	−50	40	−6	4.47	44	40	−14	2.88
	−52	40	−2	4.42				
Inf. frontal gyrus, pars triangularis					56	24	2	3.62
					58	22	8	3.40
Inf. frontal gyrus, pars opercularis	−56	16	12	3.15	60	14	4	3.55
					60	18	8	3.31
Precentral gyrus	−44	−6	40	3.93				
	−46	−4	36	3.72				
Postcentral gyrus	−52	−12	50	4.39				
	−48	−12	46	4.11				
Sup. parietal lobule	−38	−68	56	4.12				
Intraparietal sulcus	−46	−60	54	3.87				
Sup. temporal pole	−40	18	−24	4.49	44	20	−22	3.92
	−46	12	−20	3.92				
Sup. temporal gyrus	−62	−48	20	3.45				
Mid. temporal gyrus	−62	−48	12	4.05	66	−38	4	3.82
	−56	−44	12	3.73	66	−44	6	3.59
Hippocampus	−24	−4	−24	3.03				
	−26	−8	−24	2.99				
Mid. occipital gyrus	−40	−86	−6	3.17				
Inf. occipital gyrus	−34	−84	−12	3.98	32	−94	−6	3.18
	−32	−88	−8	3.70				
Fusiform gyrus	−32	−80	−14	3.93				
Calcarine cortex	−8	−94	−8	3.53				
	−14	−92	−2	3.50				
Lingual gyrus	−22	−88	−14	3.71				
	−12	−92	−8	3.65				
Cerebellum	−16	−84	−22	3.31	8	−74	−16	4.00
	−30	−74	−28	3.13	30	−64	−28	3.82
Pallidum	−20	0	0	3.00				
	−22	−2	−2	2.93				
**(B) GPC EFFECT**								
Mid. frontal gyrus	−38	30	30	2.98				
Mid. frontal gyrus, pars orbitalis	−26	46	−14	3.18				
Inf. frontal gyrus, pars orbitalis	−30	44	−16	3.42				
	−36	36	−4	3.22				
Inf. parietal lobule	−48	−42	40	3.28				
Fusiform gyrus	−38	−48	−18	3.25				
**(C) CONJUNCTION OF LEXICAL AND GPC EFFECTS**								
SMA	−2	10	54	5.26	8	14	52	3.37
Mid. frontal gyrus	−38	46	8	3.61	44	44	26	3.63
	−40	48	12	3.43				
Inf. frontal gyrus, pars orbitalis	−42	20	−6	5.86	50	24	−10	3.38
	−40	32	−2	4.07	48	18	−12	3.34
Inf. frontal gyrus, pars triangularis	−46	34	20	4.27				
	−46	32	16	4.25				
Inf. frontal gyrus, pars opercularis	−44	8	30	3.94				
	−48	12	28	3.92				
Precentral gyrus	−50	8	42	4.84				
	−48	6	46	4.83				
Inf. parietal lobule	−50	−50	42	3.08				
	−52	−46	42	3.02				
Sup. temporal pole	−50	14	−8	6.06				
Fusiform gyrus	−40	−64	−18	4.20				
Mid. occipital gyrus	−18	−102	0	5.80				
Inf. occipital gyrus	−28	−96	−8	4.09	24	−100	−2	4.03
	−32	−92	−10	3.17				
Calcarine cortex					18	−102	0	3.24
					20	−102	4	3.08
Cerebellum	−40	−54	−24	4.68	40	−64	−26	3.18
					34	−70	−28	3.09

#### GPC effect

##### Direct comparison approach (GPC>lexical)

An increased activation was observed in the GPC frame rather than in the lexical-semantic frame at the level of the left middle and inferior frontal gyri, of the left hippocampus, of the left inferior parietal lobule, and of the left fusiform gyrus (Table 1B).

##### Exclusive masking approach

A specific GPC effect was observed in a small subset of left-lateralized brain regions: the middle frontal gyrus, the orbital part of the inferior frontal gyrus, the inferior parietal lobule and the fusiform gyrus. (Table 2B and areas in yellow in Figure 2).

#### Conjunction of the lexical-semantic and GPC effects

The conjunction analysis revealed shared activation of the middle and inferior frontal gyri, bilaterally, of the SMA, bilaterally, of the left precentral gyrus, of the left inferior parietal lobule, of the left superior temporal pole, of the left fusiform gyrus, and the left middle occipital gyrus, of the inferior occipital gyrus, bilaterally, of the right V1 and of the cerebellum, bilaterally (Table 2C and areas in red in Figure 2).

### fMRI data: Multivariate pattern analyses (MVPA)

The first multivariate classification analysis (lexical mask) indicated that the neural pattern associated with target reading in the lexical frames represent a valid model (mean classification accuracy = 71.25%; χ^2^ = 14.5; *p* = 0.002) to correctly classify the activation patterns associated with target reading in both the lexical (28 out of 40 spmT maps correctly classified) and the sublexical frames (29 out of 40 spmT maps correctly classified).

The second multivariate classification analysis (sublexical mask) showed that the neural pattern associated with target reading in the sublexical frames represented an adequate model (mean classification accuracy = 65.00%; χ^2^ = 7.4; *p* = 0.06) to correctly classify the activation patterns associated with target reading in the sublexical frame (27 out of 40 spmT maps correctly classified), but not in the lexical frame (25 out of 40 spmT maps correctly classified).

Finally, the third multivariate classification analysis (conjunction mask) showed that the neural network activated by both the lexical and the sublexical frames did not represent a good model (mean classification accuracy = 51.25%; χ^2^ = 0.5; *p* = 0.92) to classify the activation patterns associated with target reading neither in the sublexical frame (19 out 40 spmT maps correctly classified), nor in the lexical frame (22 out 40 spmT maps correctly classified).

Recursive feature elimination further showed that within the conjunction mask there were no spatially restricted activation patterns that could distinguish between the lexical and the sublexical frames (mean classification accuracy = 52.50%; χ^2^ = 1.0; *p* = 0.80; 19/40 sublexical and 23/40 lexical spmT maps correctly classified).

## Discussion

The neural correlates of single word and pseudoword reading have been investigated in many neuroimaging studies over the past 30 years. These studies identified a left-lateralized cortical network that involved the occipito-temporal cortex, the temporal and temporo-parietal regions and the inferior frontal area, (for reviews, see Fiez and Petersen, 1998; Price, 2012). However, there is only partial agreement on the specific role of these areas in reading, and there is no conclusive evidence that favors a specific model of reading (Bergmann and Wimmer, 2008; Levy et al., 2009; Graves et al., 2010; Roux et al., 2012; Cattinelli et al., 2013).

Our attack on the fortress of the neural correlates of the dual-route model used a list-manipulation paradigm to dissociate the neurofunctional networks that underlie specific (grapheme-to-phoneme conversion, or lexical-semantic) reading procedures and that minimized the effect of stimulus type and task-demand.

We will now discuss the extent to which our behavioral and neurofunctional evidence favors dissociation between the lexical-semantic and GPC reading procedures.

### Do the frames elicit prevalent lexical-semantic rather than sublexical reading?

Significant behavioral differences emerged in reading speed between disyllabic words in the irregular word frame and disyllabic words in the pseudoword frame. This result is compatible with lexical-semantic facilitation in one frame, or with a decrement that is related to the prevalent use of the GPC procedure in the pseudoword frame, or with a combination of the two effects.

There would still be disagreement about whether a real facilitation was observed for reading in the lexical frame if one had only the pseudoword frame data as a reference point. However, comparison with an additional baseline measure (disyllabic word reading outside any frame) resulted in the observation that reading lists of target words outside any filler frame is associated with faster reading times. This result can be interpreted in different ways. An explanation might be that participants, when consistently reading the target disyllabic words, become attuned to that word length/orthography while not being *disturbed* by the fillers that come from a different orthography or by trisyllabic words. A more interesting interpretation is that reading the target words in isolation, i.e., outside of the specifically designed filler lists, is accomplished by using all possible strategies, including the sublexical and the lexical-semantic routes. By the same line of reasoning, one can assume that the comparatively longer reaction times for the stimuli in the lexical-semantic filler frame might be due to the prevalent use of a lexical-semantic strategy with relative suppression of the sublexical procedure. This was the effect that we sought with our experimental manipulations.

To summarize, our behavioral results suggest that the word-list manipulations forced participants to emphasize the sublexical GPC procedure in one condition and the lexical-semantic procedure in the other condition. However, this would not lead to reading times that are as fast as those of the “reading-in-isolation” condition, in which participants can let the two non-conflicting procedures (the two horses of the horse-race metaphor, Paap and Noel, 1991) of the dual-route model run freely.

### Lexical-semantic and sublexical networks: Univariate and multivariate analyses

In the fMRI study, we investigated the neural correlates of lexical and sublexical reading procedures using a list-manipulation paradigm. It is worth emphasizing that the BOLD signal was always associated with reading disyllabic words dispersed in the two different frames. To verify whether differences between lexical and sublexical frames may depend on the frame type, a univariate interaction analysis was firstly implemented.

No significant interaction effects emerged from the univariate analysis, suggesting that the functional anatomical differences that are elicited by either the lexical or the sublexical frame should be interpreted as favoring the route-emphasis hypothesis rather than the time-criterion hypothesis. Instead, at a behavioral level, we have no direct evidence for this hypothesis because participants were reading a set of words only in one of the lexical-semantic frame condition, in one of the sublexical condition and in the blocked condition. However, the absence of behavioral differences between similar frames, when random slopes for the participants were included in the mixed-effects model, could provide indirect evidence that the observed effects were not conditioned by a recruitment bias.

#### Lexical-semantic procedure

Reading a disyllabic word in a lexical frame activated a specific bilateral set of lexical-semantic regions, specifically the left occipital areas (BA18/19), the posterior part of the middle temporal gyri, the left temporal pole, and the dorsal portion of the left inferior parietal lobule. As confirmed by the multivariate classification analysis, this network represents a valid model to classify the haemodynamic response associated with target reading in both frames. This result supports the hypothesis that these areas are associated with the lexical-semantic procedure of reading.

As reported in literature, the lateral temporal cortex and the posterior portion of the left middle temporal gyrus are often involved in lexical-semantic processing (Vigneau et al., 2006; Binder et al., 2009; Visser et al., 2010). Significant activation of the left posterior temporal and left parietal regions have indeed been reported for irregular words compared with regular words (Frost et al., 2005; Lee et al., 2005; Senaha et al., 2005), for familiar words compared with pseudowords (Fiebach et al., 2002; Ischebeck et al., 2004; Borowsky et al., 2006), and during semantic tasks compared with phonological decision tasks (McDermott et al., 2003; Mechelli et al., 2005; Booth et al., 2006; see Price, 2012 for a review). Cattinelli et al. (2013) and Taylor et al. (2013) reported the involvement of the left middle temporal cortex in semantic processing that is consistent with these data. In particular, Taylor et al. (2013), in an attempt to clarify the relationship between functional anatomical data of both reading and cognitive models, have suggested that the posterior portion of the left middle temporal gyrus and the angular gyrus would be associated with the phonological lexical and semantic processing.

Our data demonstrate that these cerebral areas are specifically activated during reading through the lexical-semantic procedure and that their activation is independent of such factors as word frequency and imageability. The specific activation of the dorsal portion of the left inferior parietal lobule^3^ (together with the activation of the angular and supramarginal gyri), during disyllabic reading in the lexical frame also speaks in favor of an association of this area with the lexical-semantic reading procedure. Partially in line with this result, Taylor et al. (2013) reported a word > pseudoword activation cluster in the left angular and middle temporal gyri, suggesting that this pattern could reflect the engagement (via the orthographic lexicon) of either the phonological lexical or the semantic processing.

Finally, the activation of the left occipital and of the posterior occipito-temporal cortex during disyllabic reading in the lexical frame suggests that there is also preferential processing of words in the early visual areas. This result is consistent with the increased activation observed in the lingual gyrus, which has been interpreted as reflecting the engagement of global shape processing (Mechelli et al., 2000). On the contrary, neither Cattinelli et al. (2013) nor Taylor et al. (2013) observed left occipital- and posterior fusiform-specific activation for words.

#### Sublexical procedure

Results of univariate analyses suggest that the left fusiform, the left inferior parietal and the frontal cortex are specifically involved in sublexical reading. As confirmed by the multivariate classification analysis, these cerebral areas, together with the inferior parietal lobule, represent a good model to classify the haemodynamic response associated with target reading in the sublexical frame. This result suggests that these areas are associated with the sublexical reading procedure.

Notwithstanding a little ventral portion of the fusiform gyrus (x = −38; y = −48; z = −18), near to the so-called Visual Word Form Area (VWFA), was activated during disyllabic reading in the sublexical frame, the larger part of this region was activated for reading in both frames (see below for discussion).

Our data also showed that different parietal areas could be associated with different reading procedures. Similar results also emerged in a recent meta-analysis performed by Cattinelli et al. (2013). In particular, our present data show that the left inferior parietal lobule is specifically activated during word reading in the sublexical frame. Consistently with the results obtained by Taylor et al. (2013), the inferior parietal cortex appears to be involved in GPC.

It is worthy to note that the list-manipulation paradigm employed in the present study allowed us to discriminate between the specific neural effects of lexical and sublexical reading and the neural effects that are associated with such linguistic variables as word frequency and imageability, which clearly differ between words and non-words.

#### Input and output components of the reading process

The dual-route models that describe the lexical-semantic and sublexical processes as two independent paths predict that some processing units are located upstream and some downstream of the two routes and are shared by both early visual/orthographic input processing and a phonological output buffer. Our results are compatible with this hypothesis. Some brain regions were indeed activated commonly by both the lexical and the sublexical frames. In line with the univariate analyses, the multivariate classification analysis showed that this commonality network does not represent a good model to classify the haemodynamic response associated with target reading either in the lexical or in the sublexical frame. Even spatially more restricted sub-components of the commonality network did not yield successful classification of the lexical vs. the sublexical frames, as shown by recursive feature elimination. Thus, the conjunction brain areas were most likely associated with either early input or late output processes.

With regard to the input visual/orthographic processing in particular, common activation was observed at the level of the left middle occipital cortex and of the left ventral occipito-temporal area, including the so-called Visual Word Form Area (Cohen et al., 2002). Consistent with the dual-route model, the early visual analysis of written words can be described along three steps, which are letter identification, letter position encoding and letter-to-word binding. A deficit in one of these processing stages could cause letter-by-letter dyslexia/pure alexia, which is often associated with a lesion in the left ventral occipito-temporal area (Behrmann et al., 1998; Cuetos and Ellis, 1999; Cohen et al., 2003), and positional dyslexia, which has been associated with a lesion in the occipito-parietal cortex (Friedmann and Gvion, 2001). Additionally, Taylor et al. (2013) reported involvement of the left posterior fusiform and occipito-temporal cortex in non-lexical orthographic processing, which corresponds at a cognitive level to the initial analyses of letter units that are hypothesized by the DRC model. Another interpretation of the activation that emerged in the left ventral occipito-temporal areas for reading in both a lexical and sublexical frame could be termed as an “*orthographic representation matching process”* (Schurz et al., 2010), in which, in the case of words, a visual input is matched with a specific orthographic representation or in the case of pseudowords, there would be activation of multiple word representations that only partially match visual input^4^.

Our data do not allow us to distinguish between these two hypotheses.

Finally, the premotor cortex, the SMA, the left inferior frontal cortex that extend to the anterior part of the left insula and the left prefrontal cortex were commonly activated by both the lexical and the sublexical frames and seem to be associated with the phonological output buffer, i.e., the output store that would support phonological assembling and its interface to covert articulatory plans (see Price, 2012 for a review). In particular, the opercular portion of the left inferior frontal gyrus (LoIFG) is usually considered to be crucial for the reading processes. Neuroimaging studies indicate that the LoIFG is activated more strongly during phonological than during semantic decision tasks for written stimuli (McDermott et al., 2003; Mechelli et al., 2005; Booth et al., 2006), during pseudoword reading than during word reading (Fiebach et al., 2002; Mechelli et al., 2003; Borowsky et al., 2006), and during unfamiliar-word than during familiar-word reading (Fiebach et al., 2002; Ischebeck et al., 2004; Price, 2012). There is convergent evidence from patients with LoIFG lesions, who are impaired in reading pseudowords and low-frequency irregular words (Wagner et al., 2001; Fiez et al., 2006). Cattinelli et al. (2013) suggested that there is an involvement of the LoIFG in more general phonological processing and labeled the LoIFG as an area that is “sensitive to the computational load required by the reading task, rather than to any psycholinguistic variable” and/or processing units (Cattinelli et al., 2013, p. 16). However, while the present data are consistent with the assumption that the LoIFG constitutes a hub of phonological output processes (Taylor et al., 2013), Cattinelli et al.'s (2013) interpretation was further spelled out in terms of difficulty of phonological retrieval in the orthography-to-phonology conversion.

### Conclusions

The present results provide evidence of shared and divergent neural substrates for the lexical- semantic and the sublexical procedures that underlie word and pseudoword reading. The present results are based on a list-context manipulation and are not confounded by such unbalanced psycholinguistic factors as word frequency and imageability. The list-manipulation procedure may be further exploited to test cross-cultural differences in reading strategies.

It is worthy to note that our study does not provide evidence that favors one particular reading model. Indeed, both the dual-route model and the triangle model predict functional anatomical differences between the two reading frames. Our results showed the existence of a neural dissociation between the lexical and sublexical reading procedures that could be represented by the lexical-semantic and sublexical pathways that are proposed in the dual-route model or by the orthography-to-phonology and the orthography-to-semantics-to-phonology pathways in the triangle model.

### Conflict of interest statement

The authors declare that the research was conducted in the absence of any commercial or financial relationships that could be construed as a potential conflict of interest.

## Footnotes

^1^The frequent co-occurrence of more general phonological deficits in phonological dyslexia or of semantic deficits in surface dyslexia has fuelled the debate about whether specific sublexical or lexical reading procedures actually exist (see Patterson and Ralph, 1999 for a review).

^2^There are no irregularities of orthography for reading in Italian. The only reading ambiguity emerges when one has to retrieve the proper stress position for words of more than two syllables. The stress position for such multisyllabic words can be retrieved only by lexical identification and not by means of orthographic cues.

^3^The plot of the beta value for each condition, with zero reflecting activity during the baseline condition, showed greater positive activity in the word than in the pseudoword frame within the inferior parietal lobule (−56 −52 38); this suggests the absence of deactivation effects.

^4^Interestingly, the visual neurocomputational Hmax model (Riesenhuber and Poggio, 2002) for object recognition predicts that a sparse representation (fewer units) should be observed for more finely tuned neural representations, and a non-sparse representation (more units) should be observed for more broadly tuned neural representations. Considering orthographic processing, this model is in line with the assumption that the left occipito-temporal cortex contains neurons tightly tuned to whole words as result of past visual experience with them. Neurons that show high selectivity to a specific word, also show some response to other orthographically similar real words, and to similar pseudowords, thus leading to a total neural signal for pseudowords that might be equal to or even greater than that evoked by real words (Glezer et al., 2009).

## Appendix 1

**Table A1:** **List of the written items used in the study**.

**Lexical frame**		**Sublexical frame**	
**Targets**	**Fillers**	**Targets**	**Fillers**
**SESSION 1**			
LAVA	PRIVACY	FETO	VECHENDA
GOLA	LEADER	NASO	SMARILLE
SALA	SHUTTLE	RIGA	CREMPE
MOLO	STEWARD	LUPO	CIRBAGO
RUPE	DISCOUNT	TORO	FOSESCHI
NIDO	TAILLEUR	FILO	CIMPELTE
NANO	COPYRIGHT	FATA	CRUFFELE
RIVA	ZOOM	PIPA	SMETINGA
VENA	POIS	VINO	TENARGE
FUNE	JEANS	VOTO	CHITE
TOPO	TOAST	SETA	GUETA
RISO	FICTION	RAME	GELCA
BUCO	DEEJAY	CANE	OMALIRTO
PELO	BOUQUET	NOCE	COTRENCA
MINA	AUDIENCE	TUTA	APELIARO
PANE	BARBECUE	TIFO	BLACOTA
NAVE	COIFFEUR	LIDO	FASIENE
LAMA	OUTLET	VELA	AOLE
SEME	BOUTIQUE	RANA	DRIMA
MULO	DOWNLOAD	FOCE	ACENPE
	BRIOCHE		CIORGESE
	CROISSANT		FLEDA
	BLACKOUT		BILESTRO
	MOUSE		PRIELI
	YACHT		CLEFO
	BORDEAUX		BURPANTA
	BEIGE		FALTODO
	CLOWN		BRADOLLI
	COMPUTER		SCINEDA
	ROULETTE		ERLA
**SESSION 2**			
FAVA	MERITO	NODO	COGUNE
ROGO	SEDANO	RUGA	NUSERO
LOBO	TAVOLO	FOTO	SAGATO
FARO	CODICE	PERA	TESOLE
FOCA	BIBITA	FUSO	MIFURO
MELA	FEGATO	SEDE	MAPITO
VISO	REGINA	MIMO	SEFOLO
TUBO	GELATO	VELO	PATOMA
ROSA	VIGILE	LANA	VANOLE
DIVA	DECINA	MURO	FULURA
SALE	CAROTA	TANA	MIRICO
CAVO	METANO	PEPE	SATOME
CORO	PAGINA	MAGO	GETERE
LINO	PATATA	RENE	DOGORE
CODA	NATALE	TELA	POROLO
NEVE	PIRATA	RITO	VIGOTA
SUGO	REGOLA	NUCA	DILEPA
RAMO	CARICA	MUSO	DESARO
LUME	RECITA	FORO	VECATA
VASO	VISITA	DITO	MOPAVO
	COLORE		MUPICA
	LIMITE		CAFEMA
	RESINA		POCORE
	DEBITO		POLEGE
	REGIME		SANUTE
	SENATO		TICOLO
	MINUTO		SEMATA
	MATITA		LISORO
	CUCINA		FEMERA
	RAPINA		CANERA

## Appendix 2

**Table A2:** **Brain regions significantly activated during both word and pseudoword reading in Danelli et al. (2013) and used as an explicit mask in the random-effect ANOVA**.

**Brain regions**	***MNI coordinates***							
	**Left hemisphere**				**Right hemisphere**			
	***x***	***y***	***z***	***Z-score***	***x***	***y***	***z***	***Z-score***
Sup. frontal med. gyrus	−4	30	52	3.2				
Mid. frontal gyrus					52	14	48	3.3
Inf. frontal orb. gyrus	−44	30	−2	7.0	44	32	−14	5.8
	−40	28	−6	7.0	52	32	−12	5.3
Inf. frontal tri. gyrus					58	28	14	3.9
					62	22	22	4.7
Inf. frontal op. gyrus	−50	16	6	6.1				
	−50	16	20	7.2				
SMA	−6	16	48	4.7				
	−2	6	60	3.4				
Precentral gyrus	−42	2	34	5.7	46	10	48	3.2
	−44	0	54	5.5	48	10	44	3.1
Inf. parietal lobule	−54	−46	54	5.6				
Sup. temporal pole	−50	16	−18	5.8				
Mid. temporal pole					50	16	−26	4.5
Sup. temporal gyrus	−54	−46	20	4.2				
Mid. temporal gyrus	−64	−38	−2	6.5	62	−36	−4	4.8
	−56	−22	−12	4.8	64	−34	−10	4.7
Mid. occipital gyrus	−26	−98	−6	Inf				
Parahippocampal gyrus	−28	−4	−26	4.5				
	−28	−24	−18	4.4				
Inf. occipital gyrus					24	−100	−2	Inf
					32	−90	−10	5.9
Calcarine fissure					4	−82	8	3.2
					8	−84	10	3.2
Vermis					6	−78	−24	4.3
Cerebellum	−44	−54	−24	Inf	34	−76	−22	4.3
	−40	−70	−20	6.6				
